# Genotyping by sequencing reveals lack of local genetic structure between two German *Ips typographus* L. populations

**DOI:** 10.48130/FR-2022-0001

**Published:** 2022-01-26

**Authors:** Markus Müller, Mathias Niesar, Ignaz Berens, Oliver Gailing

**Affiliations:** 1 Forest Genetics and Forest Tree Breeding, Faculty for Forest Sciences and Forest Ecology, University of Göttingen, Büsgenweg 2, 37077 Göttingen, Germany; 2 Center for Integrated Breeding Research (CiBreed), University of Goettingen, 37073 Göttingen, Germany; 3 Landesbetrieb Wald und Holz NRW, Team Forest and Climate Protection, Steinmüllerallee 13, 51643 Gummersbach, Germany; 4 Landesbetrieb Wald und Holz NRW, Nationalparkforstamt Eifel, Urftseestraße 34, 53937 Schleiden

**Keywords:** GBS, Genome-wide, Coleoptera, Bark beetle, Pest, Forest, Genetics

## Abstract

The European spruce bark beetle (*Ips typographus* L.) is a serious pest in Norway spruce stands. While usually attacking freshly fallen trees or trees with a reduced defense system, also healthy trees can be infested during massive outbreaks of *I. typographus* that can occur after catastrophic events such as drought periods or storms. Knowledge of the genetic structure of this species, especially on local scales is still ambiguous. While local population structure was reported in some studies, others did not detect any differentiation among *I. typographus* populations. Here, we used genotyping by sequencing to infer the genetic structure of two *I. typographus* populations in western Germany, which had a distance of approx. 58 km from each other. Based on 16,830 SNPs we detected high genetic diversity, but very low genetic differentiation between the populations (F_ST_: 0.001) and a lack of population structure. These results suggest a high dispersal ability of *I. typographus*.

## INTRODUCTION

The European spruce bark beetle (*Ips typographus* L.) is regarded as a keystone species in forest ecosystems driving forest regeneration^[1,2]^. At the same time, it is a serious pest in Norway spruce stands (*Picea abies* [L.] KARST.)^[3]^. Usually, *I. typographus* attacks freshly fallen spruce trees or trees that have a reduced defense system due to stress^[4]^, but under massive outbreaks it can also attack healthy trees^[3,5]^. Massive population increases can occur after events such as drought periods, storms or clear cuts, and can lead to heavy losses of spruce tree stands. Therefore, knowledge of population dynamics and dispersal distances, reflected in genetic structures, are needed to inform forest management and mitigation strategies.

Several studies have analyzed the genetic structure of *I. typographus* populations using different genetic markers such as simple sequence repeats (SSRs)^[4,6−8]^, mitochondrial markers^[5,7,9,10]^, nuclear coding gene fragments^[5]^, or ribosomal DNA (internal transcribed spacer (ITS))^[9]^. These studies, however, came to different conclusions. For instance, Sallé et al.^[6]^ did not find population structure among *I. typographus* populations in Europe based on SSRs, while Mayer et al.^[5]^ detected, based on a wider sampling and mitochondrial and nuclear coding gene fragments, a geographic subdivision into a northern and southern group of this species. On a more local scale, Krascsenitsová et al.^[10]^ detected only slight genetic structure, but differences in haplotype distribution between Western/Southern Carpathians and the Eastern Carpathians using a mitochondrial marker, whereas Némethy et al.^[8]^ detected no population structure of this species in the Carpathians based on SSRs. Using the same marker type, Montano et al.^[7]^ detected population structure between *I. typographus* populations from managed and unmanaged spruce stands in the Bohemian forest and the Limestone Alps. In contrast, Gugerli et al.^[4]^ reported a lack of local population structure among *I. typographus* populations in Switzerland. Thus, especially on the local scale, the extent of population structure in this species is not well known.

The development of high-throughput-sequencing (HTS) makes it now possible to investigate genome-wide data even in non-model species. For instance, Dowle et al.^[11]^ used double-digest restriction-associated DNA (ddRAD) sequencing to investigate phylogeography and environmental adaptation in mountain pine beetle (*Dendroctonus ponderosae* Hopkins) populations across the entire distribution range of this species in western North America. HTS may also reveal a clearer pattern of population structure in *I. typographus*, but despite the recently published genome of *I. typographus*^[12]^ and antennal transcriptome studies investigating chemosensation^[13,14]^, there have been, to our knowledge, no studies conducted analyzing genome-wide genetic variation in this species. Here, we applied genotyping by sequencing of pooled samples to identify genome-wide SNPs (single nucleotide polymorphisms) in *I. typographus*, and used these SNPs to infer population structure between two *I. typographus* populations in Germany. We hypothesize that a genome-wide marker set including potentially adaptive SNPs would reveal more distinct population structure compared to previously used marker sets from more restricted parts of the genome.

## RESULTS

### Genotyping by sequencing and SNP calling

Sequencing revealed 630 million reads, which are ~10 million reads per pool. In total, 794,341 SNPs were identified across all pools. The initial filtering step (total number of fully covered SNPs in 10% of pools, MAF ≥ 0.05, min. read count of 8) led to 321,562 SNPs. Further filtering for a higher call rate (0.8), and linkage disequilibrium (R^2^ < 0.5) reduced the number of SNPs to 29,031 and 17,748, respectively. The exclusion of the population Engelskirchen due to an unknown number of sampled trees reduced the SNP number to 17,717. In total, 664 out of 11,225 cluster reference sequences, in which the SNPs were located, could not be assigned to the *I. typographus* genome (see Materials and Methods). SNPs located in these sequences were removed (in total 887 SNPs) leading to a final SNP set of 16,830 SNPs.

### Genetic diversity and differentiation

Observed heterozygosity (H_o_) was 0.245 in Ahlefeld and 0.258 in Arnsberg (Table 1). Expected heterozygosity (H_e_) was 0.265 in Ahlefeld and 0.275 in Arnsberg, and allelic richness (Ar) was 1.84 in Ahlefeld and 1.83 in Arnsberg. The inbreeding coefficients (F_is_ Ahlefeld: 0.077, F_is_ Arnsberg: 0.061) were not significantly different from zero in the two populations. The genetic differentiation between populations was very low (F_ST_: 0.001) and not significant (Table 1).

**Table 1:** Genetic diversity indices and genetic differentiation of the populations.

Population	N	H_o_	H_e_	Ar	F_is_	F_ST_
Ahlefeld	21	0.245	0.265	1.84	0.077	0.001
Arnsberg	14	0.258	0.275	1.83	0.061	
Over all	35	0.241	0.259	1.83	0.069	
N: number of pools, H_o_: observed heterozygosity, H_e_: expected heterozygosity, F_is_: inbreeding coefficient (not significantly different from zero), F_ST_: fixation index (not significant)						

The genetic diversity of the pools measured as observed heterozygosity (H_o_) was very similar and ranged from 0.227 to 0.250 (Supplemental Table S1). Also, the pairwise genetic distances among individual pools were very similar (Supplemental Table S2), and of the same magnitude among pools within populations as well as between populations (mean Hamming distance of pools both within populations and between populations: 0.2).

The AMOVA revealed that 99.92% of the variation can be found within populations and 0.08% among populations.

**Figure 1:**
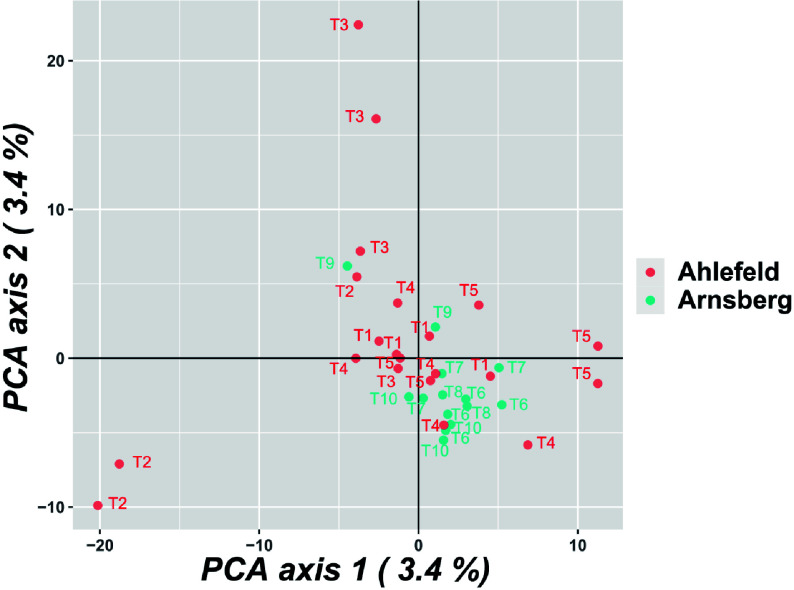
Principle component analysis (PCA) of the pools. Similar numbers refer to pools of samples taken from the same tree.

Principal component analysis (PCA) did not detect principal components (PCs) that explain a large amount of variance. The first and second PCs explain both 3.4% of the variance. The populations were not clearly separated in the PCA, and pools taken from the same tree were not more similar compared to pools taken from different trees (Fig. 1). Similar results were obtained for the neighbor joining dendrogram (Supplemental Fig. S1).

The MaxMean K method^[15]^ revealed K = 2 as the most likely number of clusters, while the Δ *K* method^[16]^ revealed K = 3. All other methods (ln Pr(XǀK)^[17]^, MedMed K, MedMean K, and MaxMed K^[15]^) revealed K = 1 as the most likely number of clusters (Supplemental Fig. S2). No distinct cluster assignment was found for the two populations, but the population Arnsberg showed a higher proportion of the blue cluster than the population Ahlefeld, when assuming K = 2 (Supplemental Fig. S3). Nevertheless, the genetic differentiation of the clusters was very low (net nucleotide distance assuming two clusters: 0.0002). Thus, there is likely no structure between the two populations (K = 1).

Of the three applied programs for the detection of outliers (BayeScan, OutFLANK, and Arlequin), only Arlequin detected three outlier loci (SNPs '54442-930_229', '45651-1144_76', and '45292-1156_123') located in sequences (GenBank accession numbers JADDUH010000001.1, JADDUH010000006.1, and JADDUH010000010.1) within contigs 1, 6, and 10 of the *I. typographus* genome^[12]^. Only for the surrounding sequence of SNP '54442-930_229' an annotation was obtained (hypothetical protein YQE_03355, partial [*Dendroctonus ponderosae*]).

## DISCUSSION

The overall observed (H_o_) and expected heterozygosity (H_e_) of the populations was 0.241 and 0.259, respectively. Since, to our knowledge, there are no other diversity data based on SNPs available for *I. typographus*, it is not possible to directly compare genetic diversity to other populations. Studies based on genome-wide SNP data of other Coleoptera species revealed, for instance, values of 0.111 (H_o_) and 0.257 (H_e_) for the Japanese rhinoceros beetle (*Trypoxylus dichotomus* L.)^[18]^, 0.078 (H_o_) and 0.087 (H_e_) for the invasive lady beetle *Harmonia axyridis* Pall.^[19]^, and 0.162 (H_o_) and 0,180 (H_e_) for the mountain pine beetle *Dendroctonus ponderosae* Hopkins^[20]^. There are more studies available that used SSR markers for the estimation of genetic diversity in *I. typographus* populations, in which higher values of diversity indices are expected compared to SNPs, due to the higher number of alleles usually present at SSR loci. For instance, Gugerli et al.^[4]^ reported values of H_e_ ranging from 0.463 to 0.560, Montano et al.^[7]^ reported values ranging from 0.387 to 0.469, and Némethy et al.^[8]^ found a mean value of H_e_ of 0.687 among populations. Thus, the genetic diversity of *I. typographus* populations seems to be comparatively high. The inbreeding coefficient (F_is_) was not significantly different from zero, hence there are no indications of homo- or heterozygosity excesses in the populations. We further found very low population differentiation (F_ST_: 0.001) in our study and a lack of population structure. These results are in agreement with other studies that analyzed population differentiation of *I. typographus* based on SSR markers on a local scale^[4,8,10]^. Only Montano et al.^[7]^ detected population structure between *I. typographus* populations from managed and unmanaged spruce stands in the Bohemian forest and the Limestone Alps. Thus, in contrast to our hypothesis, even the use of a genome-wide marker set involving potentially adaptive genetic variation did not reveal any population structure between populations. Two of three programs used for the detection of outlier loci (BayeScan^[21]^, OutFLANK^[22]^, and Arlequin^[23]^) did not reveal any outliers. Only Arlequin detected three outlier SNPs (SNPs '54442-930_229', '45651-1144_76', and '45292-1156_123'), which were located in the contigs 1, 6, and 10 of the *I. typographus* genome^[12]^. Since only two populations were compared in our study, F_ST_-heterozygosity outlier methods as implemented in Arlequin may not perform well (instead BayeScan should be suitable)^[24]^. Therefore, the outlier loci revealed by Arlequin in this study may be false positive ones.

Our results indicate a high connectivity of the populations and random mating. Indeed, a high dispersal ability of *I. typographus* is assumed^[4,6,7,25]^. Since this species is developing on weakened or recently dead trees, which are usually scarce and distributed over the landscape, it can be expected that *I. typographus* has evolved efficient foraging capacities^[6]^. Thus, wind supported dispersal distances of 43 km can be expected for this species^[25]^. Montano et al.^[7]^ even estimated a dispersal distance of more than 100 km, whereby several smaller intervening forest patches between the study areas likely helped to maintain connectivity. The distance between the populations observed in our study was approx. 58 km, and there were forest stands located in between the two study areas. Hence, it can be expected that there is migration between the two populations. Additionally, the sampling was conducted in a time of high population density of *I. typographus* in the study area. The beetles also colonized pine trees which has been observed previously^[5,26]^. We, however, did not detect genetic differences of *I. typographus* individuals inhabiting spruce or pine in our study (data not shown).

We used genotyping-by-sequencing of pooled samples in this study, since the DNA extracted from heads and legs of single beetles showed a too low quantity for sequencing. In general, pool-GBS leads to allele frequency estimates that are similar to estimates based on analysis of individuals^[27]^, but the accuracy of allele frequency estimates might be affected by unequal amounts of DNA from each individual in the pool^[28]^. Since we did not use equal amounts of DNA for pooling (tissues were pooled for DNA extraction), each individual might not have contributed in the same way to the final pool. Nevertheless, we sequenced several pools per population and the pools showed very similar diversities (Supplemental Table S1). Therefore, we assume that the pooling did not strongly affect the results of our study.

## CONCLUSIONS

We used GBS to investigate the genetic structure between two *I. typographus* populations in western Germany. We found high genetic diversity of the analyzed populations, but very low population differentiation. These results suggest a high dispersal ability of the European spruce bark beetle. The set of 16,830 SNPs provided in this study can be used in future studies of *I. typographus*. In the future, more populations spanning larger areas may be sampled to detect genomic signatures of selection. Further, environmental variables could be jointly investigated with the genomic data to conduct environmental association studies.

## MATERIALS AND METHODS

### Sampling

In three populations (Ahlefeld, Arnsberg, and Engelskirchen) located in the German federal state North Rhine-Westphalia, spruce bark beetles were sampled from standing and lying trees in 2020. In Ahlefeld and Arnsberg five trees each were sampled, whereas an unknown number of trees were sampled in the population Engelskirchen (Table 2). Since the exact number of trees sampled in Engelskirchen is unknown and the beetles of all samples were mixed in this population, samples of the Engelskirchen population were only used for SNP identification, but not for population genetic analysis. The beetles were directly sampled into 80% EtOH or first frozen and subsequently conserved in 80% EtOH.

### DNA extraction

To avoid negative effects of gut content on the sequencing, only heads and legs of the beetles were used for DNA isolation. A first attempt of DNA isolation based on single beetles revealed too low DNA quantity for sequencing. Therefore, heads and legs of five beetles of each sample were pooled for DNA isolation, which led to a sufficient DNA quality and quantity. In total, 63 pools were sent to LGC Genomics for DNA isolation (Table 2).

**Table 2:** Overview of the sampled populations.

Population	Latitude	Longitude	No. of sampled trees	No. of pools
Engelskirchen	50.97610798	7.41474115	NA	28
Ahlefeld	50.99651943	7.55328433	5	21
Arnsberg	51.44245304	7.99021258	5	14

### Genotyping by sequencing and SNP identification

Library preparation, normalized genotyping by sequencing (nGBS^[29]^), and SNP identification was conducted by LGC Genomics. Paired-end sequencing (2 × 150 bp) was conducted on an Illumina NextSeq 550 system aiming at 10 million reads per sample. Raw sequencing reads were deposited in the NCBI Sequence Read Archive (SRA) under BioProject number PRJNA781394. Since variable alignment rates between 54.9% and 92.5% (mean 75.9%) of the pools to the *I. typographus* genome^[12]^ were observed, we decided to build a cluster reference for read mapping. Thus, after demultiplexing and quality trimming, clustering of combined reads was conducted with CD-HIT-EST v4.6.1^[30]^. This widely used program (for its use with GBS data see e.g., Garsmeur et al.^[31]^, Liber et al.^[32]^, Palumbo et al.^[33]^) sorts the sequences from long to short, whereas the longest sequence becomes the representative of the first cluster. Afterwards each sequence is compared with the representative sequences of existing clusters. If the similarity is above a given threshold, the sequence is grouped into the cluster, if the threshold is not reached, a new cluster is defined^[30]^. We allowed up to 5% differences for clustering. The reads were aligned against the cluster reference using Bowtie2 v2.2.3^[34]^. Variant discovery was conducted with Freebayes v1.0.2-16^[35]^. A first filtering of SNPs was conducted (total number of fully covered SNPs in 10% of samples (pools), MAF ≥ 0.05, min. read count of 8), and the corresponding VCF file used for further analysis (for further filtering see below).

### Data analysis

The R package vcfR v1.12.0^[36]^ was used to convert the VCF file described above into the genlight format readable by the R package dartR^[37]^. The dartR v1.8.3 package^[37]^ was used for further filtering of the SNPs regarding call rate (set to 0.8, i.e., SNPs need to be present in 80% of all samples) and linkage disequilibrium (R^2^ < 0.5). To remove potential contaminations from our SNP set (i.e., the underlying cluster reference sequences) we only kept SNPs that were located in sequences that were successfully assigned to the *I. typographus* genome. For this, we first filtered the cluster reference for sequences that contained SNPs from our SNP set using SeqKit v2.0.0^[38]^. For these sequences, blastn^[39]^ searches against the *I. typographus* genome^[12]^ were performed using Blast2Go v5.2.5^[40]^. SNPs located in sequences that were not assigned to the *I. typographus* genome were removed from our final SNP set. The final SNP set can be found in Supplemental Table S3 and the corresponding sequences in Supplemental Data File S1. The R package hierfstat v0.5-7^[41]^ was used to calculate observed heterozygosity (H_o_), expected heterozygosity (H_e_), allelic richness (Ar), inbreeding coefficient (F_is_), and fixation index (F_ST_). Confidence intervals for F_is_ and F_ST_ were calculated using 1,000 bootstraps over loci. H_o_ of single pools was calculated with dartR. PGDSpider v2.1.1.5^[42]^ was used for input file conversion, and subsequently Analysis of Molecular Variance (AMOVA) based on 1000 permutations was conducted in Arlequin v3.5.2.2^[23]^. DartR was used to conduct a principle component analysis (PCA) of the pools. A neighbor joining dendrogram based on Hamming distance and 1000 bootstrap replicates was constructed with the R package poppr v2.8.7^[43,44]^. The same R package was also used to calculate pairwise genetic distances (Hamming distance) among individual pools. Computationally intensive tasks were performed on the Rstudio server v1.4.1106^[45]^ of the Gesellschaft für wissenschaftliche Datenverarbeitung Göttingen (GWDG). STRUCTURE v2.3.4^[17]^ was used to infer population structure. The admixture model and correlated allele frequencies were used. A burn-in period of 10,000 and Markov chain Monte Carlo (MCMC) replicates of 100,000 were used. Potential clusters (*K*) from 1 to 4 were tested using 5 iterations. STRUCTURE was run on the high performance computing system of the GWDG using StrAuto v1.0^[46]^. StructureSelector^[47]^ was used to determine the most likely number of *K* based on different methods such as Δ *K*^[16]^, ln Pr(XǀK)^[17]^, and the methods proposed by Puechmaille^[15]^ MedMed K, MedMean K, MaxMed K, and MaxMean K. CLUMPAK^[48]^ was used for summation and graphical representation of the STRUCTURE results. Three different types of software were used for the detection of outlier loci between the two populations. BayeScan v2.1^[21]^ was run using default parameters including 100,000 iterations and a burn-in period of 50,000. The prior odds for the neutral model were set to 1000, and a *q*-value threshold of 10% was chosen to determine significant outliers. OutFLANK^[22]^ implemented in the R package dartR v1.8.3^[37]^ was run using default parameters. Finally, Arlequin v3.5.2.2^[23]^ was run with the non-hierarchical finite island model using 100,000 simulations and 100 simulated demes. *P*-values were adjusted using the p.adjust R function^[49]^ applying a false discovery rate (FDR) of 0.05 to determine significant outliers. Annotations for significant outlier loci were obtained by searching the relevant sequences against the NCBI non-redundant protein sequences database using BLASTX^[39]^. For all mentioned analyses in R, R v4.0.4^[49]^ was used.

## SUPPLEMENTARY DATA

Supplementary data to this article can be found online.
