# Supplementary material for: Genotyping by sequencing reveals lack of local genetic structure between two German Ips typographus L. populations
Source: For Res (Fayettev). 2022 Jan 26;2:1. doi: 10.48130/FR-2022-0001 (PMC11524269; doi:10.48130/FR-2022-0001)
Supplement: Supplementary file 1 — Supplementary data to this article can be found online. [file FR-2022-0001-S1.zip › 10.48130_FR-2022-0001-Suppl-TableS1.docx]

**Table S1** Observed heterozygosity (H_o_) of the different pools

| Pool name | H_o_ |
| --- | --- |
| T1-01 | 0.242 |
| T1-04 | 0.244 |
| T1-05 | 0.244 |
| T1-10 | 0.239 |
| T2-02 | 0.227 |
| T2-06 | 0.236 |
| T2-07 | 0.237 |
| T3-03 | 0.243 |
| T3-08 | 0.242 |
| T3-09 | 0.248 |
| T3-11 | 0.246 |
| T4-12 | 0.229 |
| T4-13 | 0.242 |
| T4-14 | 0.243 |
| T4-15 | 0.230 |
| T4-16 | 0.244 |
| T5-45 | 0.250 |
| T5-46 | 0.245 |
| T5-47 | 0.248 |
| T5-48 | 0.249 |
| T5-49 | 0.248 |
| T6-50 | 0.243 |
| T6-51 | 0.245 |
| T6-52 | 0.244 |
| T6-53 | 0.246 |
| T7-54 | 0.241 |
| T7-55 | 0.245 |
| T7-56 | 0.243 |
| T8-57 | 0.246 |
| T8-62 | 0.248 |
| T9-58 | 0.245 |
| T9-63 | 0.238 |
| T10-59 | 0.240 |
| T10-60 | 0.242 |
| T10-61 | 0.239 |
