# Supplementary material for: Genotyping by sequencing reveals lack of local genetic structure between two German Ips typographus L. populations
Source: For Res (Fayettev). 2022 Jan 26;2:1. doi: 10.48130/FR-2022-0001 (PMC11524269; doi:10.48130/FR-2022-0001)
Supplement: Supplementary file 1 — Supplementary data to this article can be found online. [file FR-2022-0001-S1.zip › 10.48130_FR-2022-0001-Suppl-FigureS1.docx]

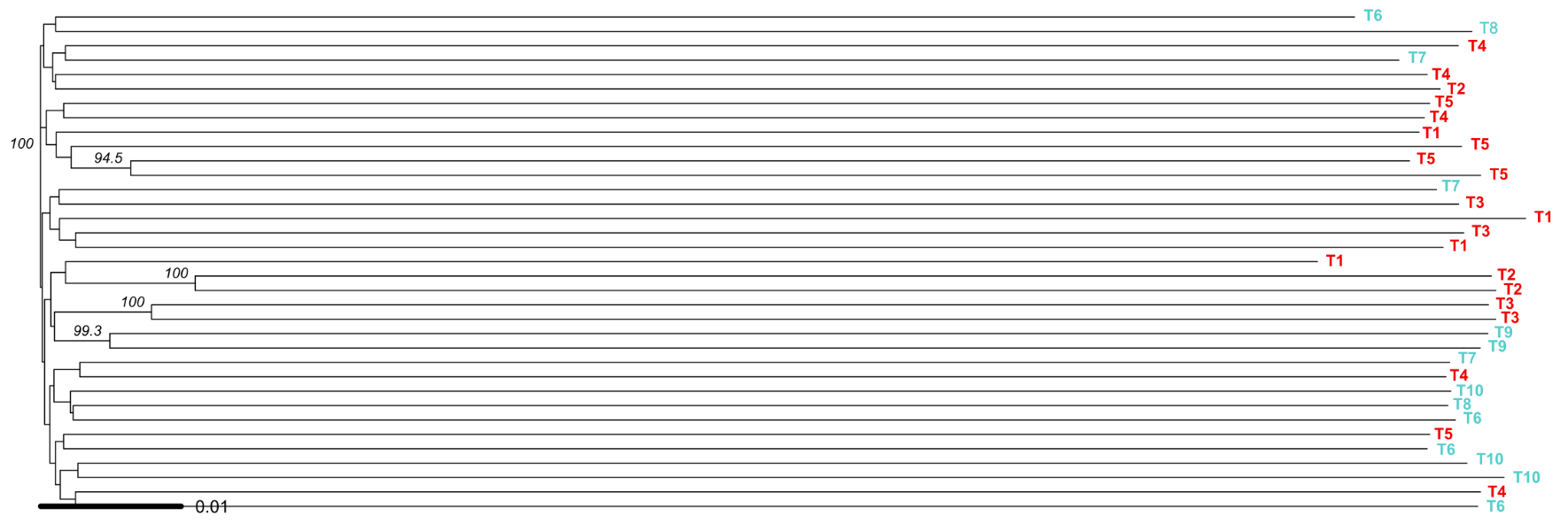


**Figure S1** Neighbor joining dendrogram for the pools of the different populations. Similar numbers refer to pools of samples taken from the same tree. Blue: population Arnsberg, red: population Ahlefeld. Bootstrapping values ≥ 50 are shown.
