# Supplementary figures and images for: Genotyping by sequencing reveals lack of local genetic structure between two German Ips typographus L. populations
Source: For Res (Fayettev). 2022 Jan 26;2:1. doi: 10.48130/FR-2022-0001 (PMC11524269; doi:10.48130/FR-2022-0001)

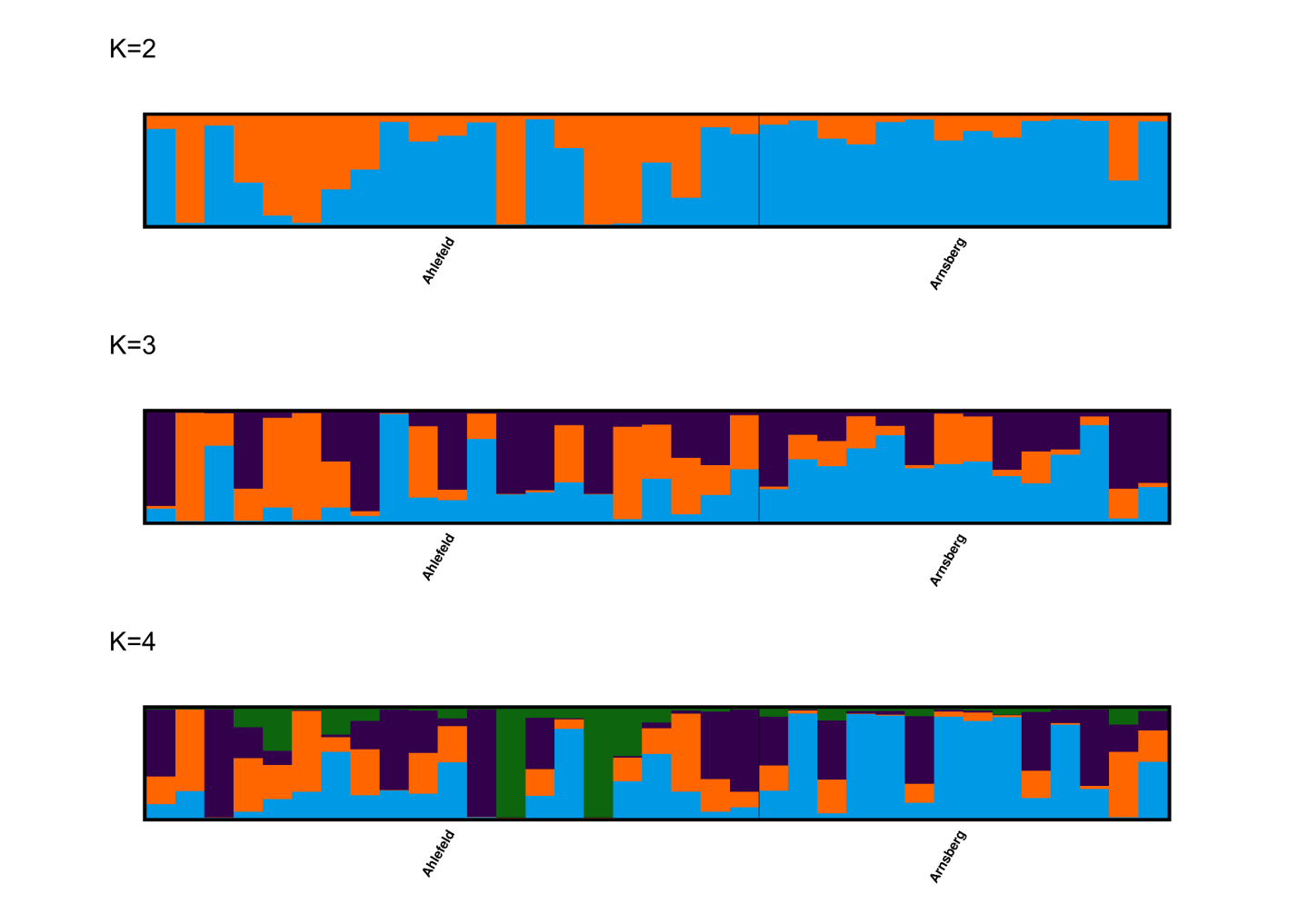


**Figure S3** Clustering of individuals for K = 2, K = 3, and K = 4.

Supplement: Supplementary file 1 — Supplementary data to this article can be found online. [file FR-2022-0001-S1.zip › 10.48130_FR-2022-0001-Suppl-FigureS3.docx]
